# Haematocrit, eggshell colouration and sexual signaling in the European starling (*Sturnus vulgaris*)

**DOI:** 10.1186/s12898-016-0084-x

**Published:** 2016-06-27

**Authors:** Raime B. Fronstin, Stephanie M. Doucet, Julian K. Christians

**Affiliations:** Department of Biological Sciences, Simon Fraser University, 8888 University Drive, Burnaby, BC V5A 1S6 Canada; Department of Biological Sciences, University of Windsor, 401 Sunset Avenue, Biology Building, Windsor, ON Canada

**Keywords:** Eggshell colour, Haematocrit, Sexual signal

## Abstract

**Background:**

One hypothesis to explain the blue–green colour of the eggs of many bird species is that it is a sexually-selected signal of the laying female’s quality, which males use to determine their investment. This hypothesis requires that eggshell pigmentation carries a cost or is otherwise linked to female quality. One potential cost is that biliverdin, a haem derivative and the pigment responsible for eggshell colouration, is limiting. To assess this potential cost, we attempted to manipulate haematocrit and haemoglobin in free-living European starlings (*Sturnus vulgaris* Linnaeus). Upon collecting unmanipulated first clutches, we treated females with phenylhydrazine (PHZ), a haemolytic agent, and measured the blue–green chroma and reproductive performance of replacement clutches. We also investigated whether eggshell colour was associated with haematocrit or haemoglobin levels in unmanipulated first clutches. To test whether eggshell colour might act as a sexual signal, we examined associations between eggshell colour and reproductive performance, as well as the provisioning rate of the male.

**Results:**

PHZ-treatment did not affect eggshell colour in replacement clutches. In unmanipulated first clutches, eggshell colour was not correlated with haematocrit or haemoglobin levels. Eggshell colour was correlated with female mass in unmanipulated first clutches but not replacement clutches. Chicks from eggs with higher eggshell colour had higher haemoglobin levels and longer tarsi just prior to fledging, suggesting that eggshell colour could reflect brood quality. However, eggshell colour was not correlated with the provisioning rate of the male or any other measure of reproductive performance.

**Conclusions:**

We found no evidence to support the hypothesis that the availability of resources required for the synthesis of pigment limits eggshell colour in European starlings, or that eggshell colour is used by males to determine their level of reproductive investment. We found little evidence that eggshell colour is correlated with female or offspring quality in this species.

**Electronic supplementary material:**

The online version of this article (doi:10.1186/s12898-016-0084-x) contains supplementary material, which is available to authorized users.

## Background

The functions of avian eggshell colour and patterning and the mechanisms underlying variation in these traits remain poorly understood [1–3]. Numerous functions of eggshell pigmentation have been proposed [2–6], and the breadth of patterns across species is likely the result of multiple functions [7]. Blue–green colouration is found in many species [3, 4] and is conspicuous to some predators, raising the question of why it is maintained [8, 9]. Among the possible explanations, one hypothesis is that the blue–green colour is a sexually-selected signal of the laying female’s quality, which males use to determine their own level of post-hatching investment [10]. This hypothesis predicts that blue–green egg colouration should be positively correlated with female quality, male provisioning effort and, consequently, the number and/or quality of offspring at fledging. Although some studies have supported these predictions [11–15] other studies have found either no or mixed support [1, 16–18].

A key assumption of the sexual-selection hypothesis is that eggshell colouration either carries a cost or is otherwise linked to female quality in such a way that the honesty of the signal is maintained [10]. One potential cost is that biliverdin, the pigment responsible for blue–green egg colouration, might be a limited resource. Biliverdin has strong antioxidant activity [19] and therefore it may be costly for mothers to allocate this pigment to eggshells if this depletes their own antioxidant supplies [10]. Consistent with this hypothesis, correlations have been observed between maternal plasma antioxidant capacity and blue–green eggshell colour [9, 12].

Biliverdin is synthesized from haem by haem oxygenase [20], and eggshell biliverdin either comes from the circulation [21–23] or de novo synthesis within the eggshell gland [24, 25]. In either case, eggshell biliverdin or the resources necessary for its production (haem or haem precursors) must come from the circulation. We attempted to experimentally manipulate haematocrit and haemoglobin, and therefore the availability of circulating resources for pigment deposition. Specifically, we treated breeding female European starlings (*Sturnus vulgaris*) with phenylhydrazine hydrochloride (PHZ), a xenobiotic oxidant known to lyse red blood cells and thereby reduce haematocrit, resulting in reduced circulating haemoglobin concentration within 24 h of treatment [26–28]. Our first prediction was that treatment with PHZ should alter the intensity of blue–green colouration. Depending on the mechanisms by which the resources for pigment deposition are obtained from the circulation, lysis of red blood cells might decrease the intensity of blue–green colouration (if the eggshell gland obtains resources for biliverdin synthesis or deposition directly from erythrocytes), or might increase colouration (if lysis increases haem or haem precursor availability in circulation). Alternatively, if the availability of circulating resources is not limiting we should find no effect of PHZ treatment. Our second prediction was that, if eggshell colouration is limited by circulating resources, then eggshell colour should be correlated with female haematology (haematocrit and haemoglobin) in unmanipulated first clutches. Our third prediction was that, if eggshell colour is a signal of female quality, variation in eggshell colouration should be related to traits that reflect female quality, male provisioning effort, and breeding success (i.e., brood quality and fledging success). Although we do not know of evidence that egg colour is a sexual signal in European starlings, previous work has suggested a role of sexual selection in the blue–green colouration of spotless starling (*Sturnus unicolor*) eggs [15].

## Methods

The present study analysed eggshell colour in 1 year (April–July 2009) of a 3-year study of a free-living nest box population of European starlings [29]. The field site is located at a dairy farm (49^o^10′N, 122^o^50′W) and consists of ~200 wooden nest boxes mounted on barns and posts (minimum height ~2 m). Nest boxes were monitored daily to establish the date of clutch initiation, laying sequence, egg size, clutch size, and the date of clutch completion for all clutches. Eggs were numbered as they were laid and were measured with calipers; mass was calculated using the formula: mass = 0.0009159 × length^0.954^ × width^1.877^ [30].

### Experimental treatment and sample collection

After completion of the first clutch, females were captured in their nest boxes just before dawn, weighed (±0.01 g), banded (permit 10,646 K from the Canadian Wildlife Service) and alternately assigned to one of two treatment groups, phenylhydrazine (PHZ, Sigma-Aldrich, Ontario Canada; n = 30) or control (n = 33). Although PHZ has been used widely to induce anemia [31] and we do not know of off-target effects unrelated to red blood cells, we cannot exclude the possibility that PHZ affected other parameters.

A pre-treatment blood sample was collected by puncturing the brachial vein with a 26½-gauge needle and collecting blood (<700 µl) into heparinized capillary tubes. A bolus injection of PHZ in saline (1.25 mg/100 g body weight in approximately 100 µl) or saline alone as the control (100 µl injection volume) was injected into the pectoral muscle. In order to induce re-laying, entire first clutches were removed prior to females being returned to their nest boxes. All eggs were stored at −20 °C until they could be analyzed for eggshell colouration (see below). Females were returned to the nest box and allowed to lay a replacement clutch without further manipulation. Daily monitoring of nests was continued to collect laying date, egg, and clutch size information for these post-treatment replacement clutches.

To avoid the risk of desertion, we did not recapture females until 1 or 2 days after completion of the replacement clutch, at which time a second blood sample was taken (PHZ, n = 17; control, n = 16). Given that the first egg of the replacement clutch was laid 8–9 days after treatment (described below), clutch size was ~5, and the second blood sample was taken 1–2 days after the completion of the replacement clutch, the time between blood samples was typically ~15 days. For comparison, haematocrit and haemoglobin return to pre-treatment levels 5–10 days post PHZ treatment, respectively, in captive non-breeding European starlings [28]. There was no desertion as a result of the second blood sample.

Beginning 9 days after clutch completion, eggs were monitored for signs of hatching (starring or pipping) and when hatching was imminent the entire clutch was removed from the nest and placed in an incubator until hatching (1–15 h). In order to maintain maternal incubation behavior, removed clutches were replaced with dummy eggs. Hatching eggs in the incubator allowed us to determine brood size at hatching and hatchling mass, and to collect eggshells from replacement clutches for further colour measurement. Hatchlings were returned to the nest immediately after measurement. During chick rearing, on day 6, 7 and 8 post-hatching (the period of most rapid chick growth), nests were observed for 30 min and maternal and paternal provisioning visits to the nest were recorded. This measure of parental care is highly correlated among successive days [32], and has revealed effects of experimental treatments in previous studies [33–35]. In order to determine chick quality just prior to fledging, at 17 days post-hatching we measured chick tarsus length, body mass and collected blood samples to obtain chick haematocrit and haemoglobin levels. Nests were then monitored for fledging and brood size at fledging was recorded.

### Haematocrit and haemoglobin measurement

Whole blood was used to measure pre-treatment (baseline) and post-treatment haematocrit levels and whole blood haemoglobin concentration. Haematocrit levels (Hct) were measured in duplicate following centrifugation at 13,000*g* for 3 min [36]. Whole blood haemoglobin (Hb, g/dl) was measured using the modified cyanomethaemoglobin method as previously described [36].

### Eggshell colour measurement

Eggshell fragments were washed with distilled water and the median part of each eggshell was measured using a USB4000 reflectance spectrometer and PX-2 light source (Ocean Optics); eggs from first clutches (collected whole) were broken to be comparable with eggs from second clutches (which were collected after hatching). We used a reflectance probe tipped with a rubber stopper that maintained the probe 3 mm from and perpendicular to the eggshell surface, and excluded external light. We collected three measurements per egg, lifting and replacing the probe between each measurement, and averaged these measurements to obtain a single reflectance spectrum per egg. We calculated blue-green chroma (hereafter BGC) as the ratio of the reflectance between 400 and 570 nm to the total reflectance between 300 and 700 nm. This measure is indicative of pigment concentration [37–40] and corresponds to the region of greatest reflectance of biliverdin [41]. Consequently, we would expect that BGC would be the main cue for male assessment of female quality [17].

### Statistical analyses

Statistical analyses were performed using SAS software version 9.2 (SAS Institute 2008). Correlations between mean BGC and female or reproductive traits were analysed using Pearson’s product moment correlations or partial correlations (CORR procedure) while differences between treatment groups were analysed using general linear models (GLM procedure). Laying interval, i.e., the time from treatment to initiation of the replacement clutch, was not normally distributed and therefore we used a Kruskal–Wallis test (NPAR1WAY procedure) to assess differences in laying interval between treatment groups. Relationships between eggshell colour and reproductive traits in replacement clutches were analyzed using a general linear model including the effects of mean BGC of the replacement clutch, treatment and the mean BGC by treatment interaction. To account for multiple chicks per female, analyses of chick traits used repeated-measures mixed linear models (MIXED procedure) with female identity included as a repeated subject effect and mean BGC of the replacement clutch, treatment and the mean BGC by treatment interaction included in the model. Power analyses were performed using the POWER procedure (onewayanova and onecorr statements).

## Results

There was no difference between treatments in the proportion of females that were observed to produce a replacement clutch (PHZ: 13/30 = 43 %; control: 16/33 = 48 %; $\chi_{1}^{2}$ = 0.17; P = 0.68); some females likely produced a replacement clutch in one of the numerous natural nesting cavities in the area and so were not observed to renest. Haematocrit, haemoglobin and mean BGC of the first clutch did not differ between females that were observed to produce a replacement clutch and those that were not (Additional file 1: Table S1). As expected, there was no difference in pre-treatment haematocrit or haemoglobin between treatment groups among females that produced a replacement clutch (Table 1). However, by chance, females assigned to the PHZ treatment had larger pre-treatment mean egg mass (F_1,27_ = 4.55, P = 0.04; PHZ 7.3 ± 0.1 g; control 6.9 ± 0.1 g). We found no difference between treatment groups in replacement clutch post-laying haematocrit (Table 1). In contrast, replacement clutch post-laying haemoglobin was affected by treatment, with PHZ-treated females having higher replacement clutch haemoglobin than controls (Table 1), which was not expected given that PHZ treatment reduces circulating haemoglobin within 24 h of treatment [28].

**Table 1:** Haematological and eggshell colour differences between treatment groups, pre- and post-treatment

Trait	N	F	Df	P	PHZ	Control	Additional terms in model
Pre-treatment Hct (%)	29	1.09	1,27	0.31	50.8 ± 1.1	49.2 ± 1.0	
Pre-treatment Hb (g/dl)	26	0.52	1,24	0.48	14.5 ± 0.6	13.9 ± 0.5	
Pre-treatment mean BGC	29	1.70	1,27	0.20	0.473 ± 0.003	0.478 ± 0.002	
Post-treatment Hct (%)	29	0.03	1,26	0.86	51.2 ± 1.1	51.5 ± 1.0	Pre-treatment Hct, F_1,26_ = 3.43, P = 0.08
Post-treatment Hb (g/dl)	26	6.89	1,23	0.015	17.3 ± 0.5	15.6 ± 0.4	Pre-treatment Hb, F_1,23_ = 7.17, P = 0.013
Post-treatment mean BGC	28	0.96	1,25	0.34	0.470 ± 0.002	0.468 ± 0.002	Pre-treatment BGC, F_1,25_ = 14.47, P = 0.0008
BGC of first egg of replacement clutch	20	0.01	1,17	0.92	0.467 ± 0.002	0.467 ± 0.002	Pre-treatment BGC, F_1,17_ = 17.44, P = 0.0006

Means are least-squares means ± standard errors from general linear models.For post-treatment traits, including laying interval in the model yielded similar results (Additional file 2: Table S2)

Laying interval was significantly greater among PHZ treated individuals (first egg of replacement clutch was laid 8–19 days after treatment, median: 9 days) compared with control individuals (first egg of replacement clutch was laid 7–11 days after treatment, median 8 days; $\chi_{1}^{2}$ = 8.63, P = 0.0033).

### Prediction 1: If nutrients for pigment synthesis are limited, PHZ-treatment should influence eggshell colouration in replacement clutches

As expected, there was no difference in pre-treatment mean BGC between treatment groups (Table 1). Treatment did not affect the mean BGC of replacement clutches (Table 1); including laying interval in the model yielded similar results (Additional file 2: Table S2). Eggs laid earlier in the laying sequence (i.e., sooner after treatment) might be expected to show a greater effect of treatment, but there was no effect of treatment on the colour of the first egg of the replacement clutch (Table 1), and no interaction between treatment and laying sequence (F_5,85_ = 0.27; P = 0.93). First clutch mean BGC was correlated to replacement clutch mean BGC among control females (*r* = 0.57, P = 0.03, n = 15), and PHZ-treated females (*r* = 0.64, P = 0.02, n = 13).

### Prediction 2: If eggshell colouration is limited by circulating resources, then eggshell colour should be correlated with female haematology in unmanipulated first clutches

In first unmanipulated clutches (including females that did not renest), mean BGC was independent of all post-laying haematological traits (Table 2).

**Table 2:** Correlations between eggshell colouration and haematological and reproductive traits in first, unmanipulated clutches

Trait	Mean BGC		
	N	R	P
Female mass	63	0.27	0.03
Haematocrit	55	−0.16	0.23
Haemoglobin	55	−0.18	0.20
Laying date	63	0.00	0.97
Mean egg mass	63	−0.01	0.95
Clutch size	63	0.01	0.97

Analyses include females that were not observed to produce a replacement clutch. For all traits except for female mass, analyses include female mass as a partial variable in the CORR procedure

### Prediction 3: If eggshell colour is a sexual signal, variation in colour should be correlated with female quality, provisioning effort, breeding success, and chick quality

**Fig. 1:**
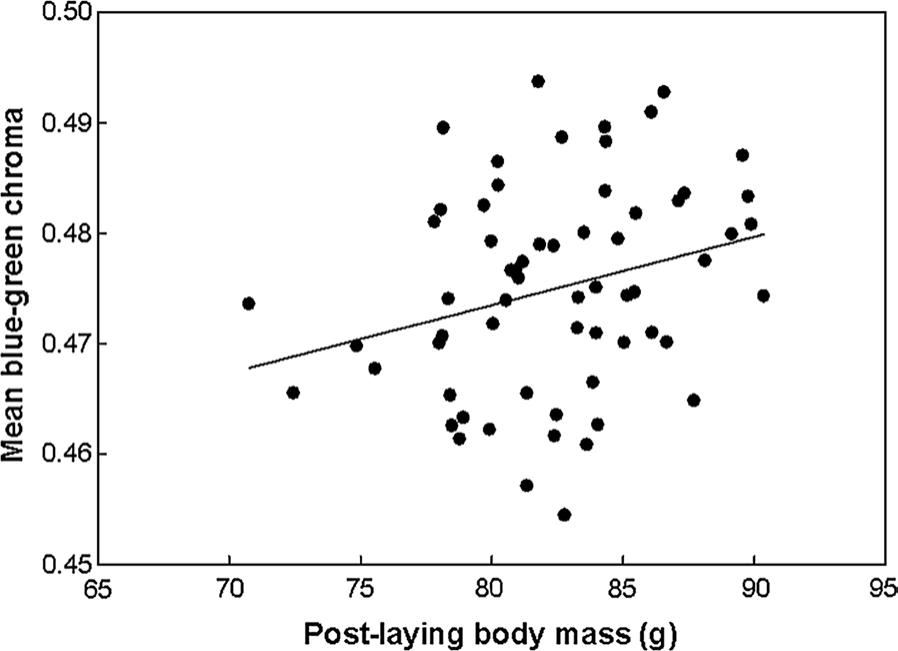
Correlation between mean blue–green chroma and female body mass within first (i.e., pre-treatment) clutches

In unmanipulated first clutches (including females that did not renest), mean BGC was positively correlated with post-laying female body mass (Table 2; Fig. 1), but was not correlated with laying date, mean egg mass, or clutch size (Table 2).

**Fig. 2:**
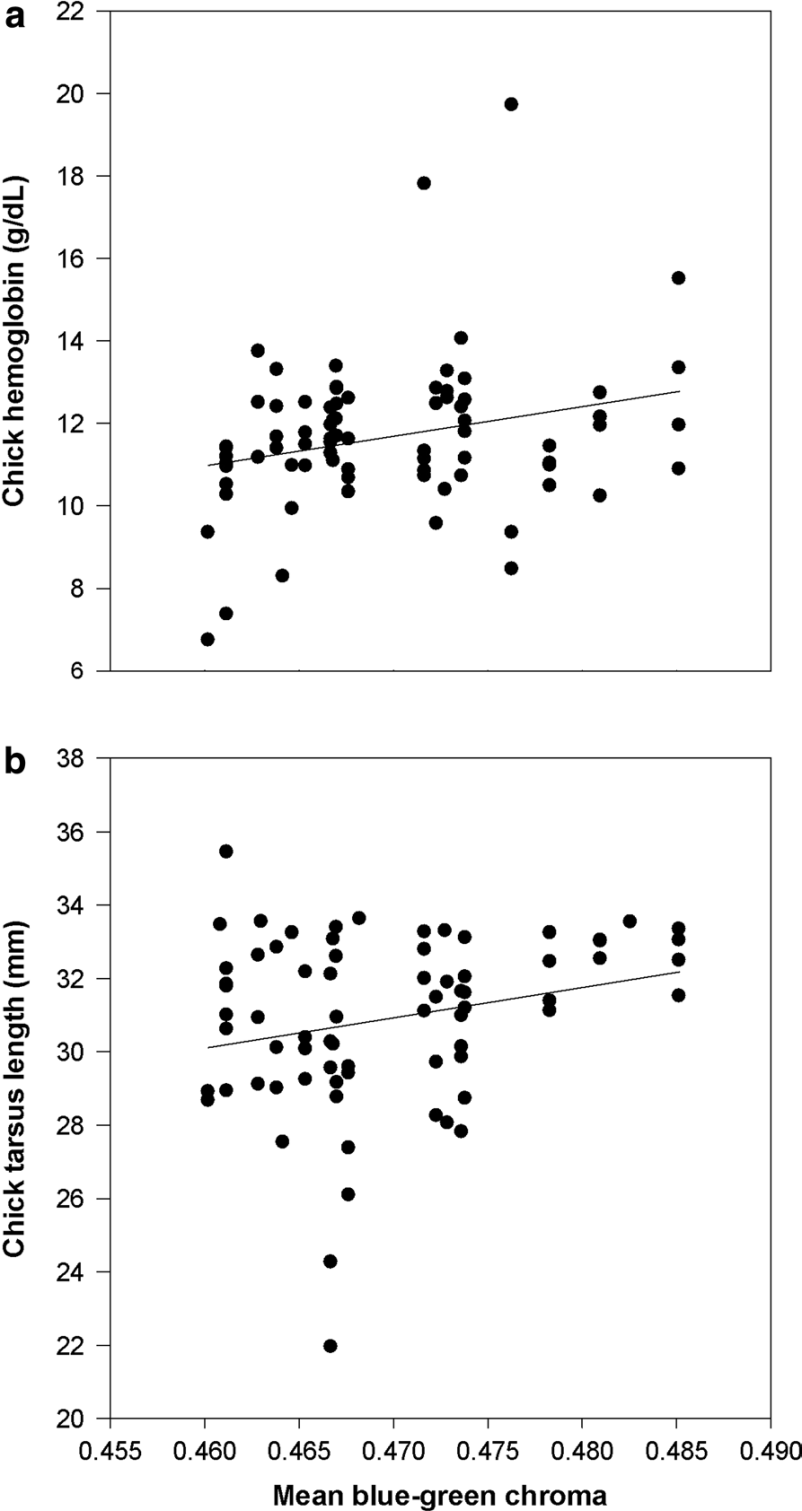
Correlation between mean blue-green chroma in replacement clutches and chick **a** haemoglobin, and **b** tarsus length

There was no relationship between mean BGC and most reproductive traits in replacement clutches (Table 3). However, chick haemoglobin and tarsus length at 17 days of age were positively related with mean BGC of the replacement clutch (Table 3; Fig. 2). There were few effects of treatment itself (Table 3), as reported previously [29].

**Table 3:** Associations between eggshell colour and reproductive performance in replacement clutches

Trait	Df	Mean BGC		Treatment	
		F	P	F	P
Interval between treatment and day first egg of replacement clutch (days)	1,25	0.00	0.96	5.53	0.03
Mean egg mass	1,25	0.38	0.54	3.26	0.08
Clutch size	1,25	1.23	0.28	1.91	0.18
Female body mass at clutch completion	1,25	0.63	0.43	0.97	0.33
Brood size at hatch	1,25	0.33	0.57	0.24	0.63
Hatchling mass^a^	1,24	0.30	0.59	3.22	0.09
Maternal provisioning (nest visits per chick)	1,23	0.63	0.44	4.58	0.04
Paternal provisioning (nest visits per chick)	1,23	0.55	0.46	0.25	0.62
Total provisioning (nest visits per chick)	1,23	0.59	0.45	2.04	0.17
Hct of 17 day old chicks^a^	1,20	1.56	0.23	8.90	0.01
Hb of 17 day old chicks^a^	1,20	5.83	0.03	1.17	0.29
Mean tarsus length of 17 day old chicks^a^	1,23	4.55	0.04	0.40	0.53
Mean mass of 17 day old chicks^1^	1,23	1.18	0.29	1.15	0.29
Brood size at fledging	1,25	0.81	0.38	0.10	0.75

Results are from general linear models. Although 29 females produced a replacement clutch, eggshells were not recovered from one replacement clutch and so sample sizes are 28 for all traits except provisioning, for which they are 26. Initial analyses included the eggshell colour * treatment interaction, but in no case was this significant (Additional file 3: Table S3), and it was therefore removed

^a^Repeated measures analysis with female identity included as a repeated subject effect. The analysis of hatchling mass included first clutch egg mass as a covariate (F_1,24_ = 76.61, P < 0.0001) since eggs were larger pre-treatment in the PHZ group

## Discussion

The sexual selection of eggshell colour hypothesis is predicated on eggshell colouration being costly or mechanistically linked to female quality. One potential cost is that biliverdin, a derivative of haem and the pigment responsible for blue–green egg colour, may be a limited resource, which would maintain the honesty of the signal. We tested three key predictions of the sexual selection of eggshell colour hypothesis in European starlings. To test our first prediction, we attempted to manipulate female haematology to examine whether the availability of circulating resources for biliverdin synthesis limits eggshell colouration. Specifically, we treated females with PHZ, which is known to lyse red-blood cells and therefore decrease haematocrit and haemoglobin. PHZ has been used widely to induce anemia [31], and while we do not know of off-target effects, these remain a possibility. Prior studies in captive non-breeding European starlings found that haematocrit and haemoglobin return to pre-treatment levels 5 to 10 days post PHZ treatment, respectively [28], although this recovery might be expected to take longer in free-living reproducing birds. Given that pigmentation of the eggshell is deposited approximately 4 h prior to laying [42, 43], the initiation of eggshell pigmentation in the replacement clutch may therefore have begun after we expected haematocrit levels to return to normal within the treatment group. Unexpectedly, total haemoglobin was significantly higher among post-treatment PHZ individuals. A possible explanation for this result is that erythropoietin production levels were increased to stimulate the synthesis of new red blood cells to return haematocrit to normal [31], and the effect on haemoglobin synthesis was greater than that on erythropoiesis. Furthermore, while haematocrit levels may have returned to control levels by the time pigment was deposited, the proportion of immature red blood cells was expected to be considerably higher than normal due to reticulocytosis, which occurs for up to 2–3 weeks after transient anemia [36, 44].

Given that haemoglobin levels were increased in the treatment group upon completion of the replacement clutch, and that treated females would have been undergoing reticulocytosis during eggshell pigmentation, we would expect our experimental manipulation to have some effect on eggshell colouration if haem or haem precursor availability in the circulation was limiting for biliverdin production for eggshell pigmentation. However, we detected no effects of PHZ treatment on eggshell colour. This result may indicate that the synthesis of biliverdin is not limited by the availability of haem to haem oxygenase, but it is also possible that the changes in haemoglobin were too small to influence biliverdin synthesis. Our results are consistent with the only other study that has attempted to manipulate female haematology to assess effects on eggshell colour. De Coster et al. [45] indirectly manipulated the numbers of immature erythrocytes in females by infecting nests with hen fleas, but found no association between parasite abundance and eggshell darkness.

Our second prediction was that eggshell would be correlated with female haematology. Considering only unmanipulated first clutches in our study, we found no correlations between eggshell colour and haematocrit or circulating haemoglobin. A limitation of this approach was that we examined associations with post-laying female mass and haematological parameters; pre-laying measures would better reflect the availability of haem and/or haem precursors during eggshell deposition. Our predictions were based on the hypothesis that the availability of haem to haem oxygenase might be a limiting step in the deposition of biliverdin into eggshells. However, biliverdin deposition might also be limited by the level of haem oxygenase expression, or the transfer of biliverdin between its sites of synthesis and deposition [50].

Our third prediction was that eggshell colour would be correlated with female quality, provisioning rate, breeding success, and chick quality. Our only evidence that eggshell colour might act as a signal of female quality was a correlation between eggshell colour and female mass in first clutches, as observed by others [38, 46]. While such an association was not observed in replacement clutches, the correlation between pre- and post-treatment eggshell colour suggests that this is a repeatable trait of females, as is circulating haemoglobin concentration. In unmanipulated first clutches, there were no associations between eggshell colour and measures of clutch quality. While we did not investigate the macro- and micronutrient composition of eggs, a previous study of European starlings found that eggshell colour was negatively related to yolk carotenoid concentration, and not related to yolk mass or lysozyme concentration [50]. Likewise, eggshell colour was not correlated with most aspects of reproductive performance (lay date, clutch size, egg mass and chick mass) in replacement clutches in our study, but was correlated with chick haemoglobin levels and tarsus length. Krist and Grim [17] found that cross-fostered chicks raised by females who laid eggs with greater eggshell BGC had longer tarsus lengths. However, there was no association between a chick’s own eggshell colour and its tarsus length. Likewise, Hanley and Doucet [16] found no correlations between eggshell colour and offspring quality.

We also found no evidence that eggshell colour was a signal used by males to determine their level of provisioning. Several studies have found positive correlations between paternal care and eggshell colour [9, 14, 15] while others have not [16, 17, 47, 48] or have found mixed support [49]. While it has been hypothesized that the blue-green colour of eggs may provide a signal to males of a female’s antioxidant capacity, biliverdin concentration explains less than half the variation in colour of European starling eggs [50].

Given that we found few effects of treatment or associations between eggshell colour and measures of reproductive performance, it is important to consider whether our analyses had substantial statistical power to detect effects and associations that might be biologically important. We calculated our power to detect an effect of the magnitude observed by Soler et al. [15] who manipulated female condition using wing feather removal and detected a reduction in eggshell colouration in a similar species, the spotless starling ; colour was reduced from 0.555 to 0.537 (Fig. 3 in [15]). Given a standard error of 0.002 (Table 1, present study), and therefore a standard deviation of 0.008, our power to detect an effect of this magnitude would be very high (0.99). Our power to detect an effect of the magnitude observed due to food supplementation (0.594 vs. 0.586; Table 1 in [38]) was lower (0.72). To detect correlations between eggshell colour and various traits, our sample sizes ranged from 55 to 63 in unmanipulated first clutches, and 26–28 in replacement clutches, giving us reasonable power (0.8 or higher) to detect associations with correlation coefficients of 0.4 and 0.5–0.55, respectively. Associations with correlation coefficients greater than 0.4 have been observed between eggshell colour and female traits [17, 51], and correlation coefficients as high as 0.64 have been reported for fledging success and egg colour [51]. Thus, the lack of effects and associations observed in the present study likely represent real differences between our system and these other studies.

## Conclusions

We found little support for the hypotheses that the availability of circulating resources for pigment synthesis limits eggshell colour, that eggshell colour reflects maternal or brood quality, or that males adjust their provisioning in response to eggshell colour in European starlings. Our findings contribute to the growing body of literature suggesting that multiple non-mutually exclusive hypotheses may explain the diversity of avian eggshell colouration.

## Additional files

10.1186/s12898-016-0084-x Traits of females that did or did not produce a replacement clutch.
10.1186/s12898-016-0084-x Differences between treatment groups, including laying interval in the model.
10.1186/s12898-016-0084-x Statistical interactions between eggshell colour and treatment.
10.1186/s12898-016-0084-x Raw data for Table 1.
10.1186/s12898-016-0084-x Raw data for Table 2.
10.1186/s12898-016-0084-x Raw data for Table 3.
10.1186/s12898-016-0084-x Raw data-laying interval.
10.1186/s12898-016-0084-x Raw data-individual eggs.
10.1186/s12898-016-0084-x Raw data-individual chicks.
10.1186/s12898-016-0084-x Raw data-first clutches.

## Abbreviations

BGC: blue–green chroma
Hb: haemoglobin
Hct: haematocrit
PHZ: phenylhydrazine hydrochloride

## References

[1] Gosler AG, Barnett PR, Reynolds SJ (2000). Inheritance and variation in eggshell patterning in the great tit Parus major. Proc R Soc Lond B Biol Sci.

[2] Maurer G, Portugal SJ, Cassey P (2011). Review: an embryo’s eye view of avian eggshell pigmentation. J Avian Biol.

[3] Underwood TJ, Sealy SG (2002). Adaptive significance of egg coloration. Oxf Ornithol Ser..

[4] Kilner RM (2006). The evolution of egg colour and patterning in birds. Biol Rev.

[5] Lahti DC (2008). Population differentiation and rapid evolution of egg color in accordance with solar radiation. Auk.

[6] Westmoreland D, Kiltie RA (1996). Egg crypsis and clutch survival in three species of blackbirds (Icteridae). Biol J Linn Soc.

[7] Hanley D, Cassey P, Doucet SM (2012). Parents, predators, parasites, and the evolution of eggshell colour in open nesting birds. Evol Ecol.

[8] Avilés JM, Soler JJ, Pérez-Contreras T (2006). Dark nests and egg colour in birds: a possible functional role of ultraviolet reflectance in egg detectability. Proc R Soc B Biol Sci..

[9] Hanley D, Heiber G, Dearborn DC (2008). Testing an assumption of the sexual-signaling hypothesis: does blue–green egg color reflect maternal antioxidant capacity. Condor..

[10] Moreno J, Osorno JL (2003). Avian egg colour and sexual selection: does eggshell pigmentation reflect female condition and genetic quality?. Ecol Lett.

[11] English PA, Montgomerie R (2010). Robin’s egg blue: does egg color influence male parental care?. Behav Ecol Sociobiol.

[12] Morales J, Velando A, Moreno J (2008). Pigment allocation to eggs decreases plasma antioxidants in a songbird. Behav Ecol Sociobiol.

[13] Moreno J, Osorno JL, Morales J, Merino S, Tomás G (2004). Egg colouration and male parental effort in the pied flycatcher Ficedula hypoleuca. J Avian Biol.

[14] Moreno J, Morales J, Lobato E, Merino S, Tomás G, Martínez-de la Puente J (2006). More colourful eggs induce a higher relative paternal investment in the pied flycatcher Ficedula hypoleuca: a cross-fostering experiment. J Avian Biol.

[15] Soler JJ, Navarro C, Contreras TP, Avilés JM, Cuervo JJ (2008). Sexually selected egg coloration in spotless starlings. Am Nat..

[16] Hanley D, Doucet S (2009). Egg coloration in ring-billed gulls (Larus delawarensis): a test of the sexual signaling hypothesis. Behav Ecol Sociobiol.

[17] Krist M, Grim T (2007). Are blue eggs a sexually selected signal of female collared flycatchers?. A cross-fostering experiment. Behav Ecol Sociobiol..

[18] López-Rull I, Celis P, Gil D (2007). Egg colour covaries with female expression of a male ornament in the spotless starling (*Sturnus unicolor*). Ethology.

[19] Kaur H, Hughes MN, Green CJ, Naughton P, Foresti R, Motterlini R (2003). Interaction of bilirubin and biliverdin with reactive nitrogen species. FEBS Lett.

[20] Maines MD (1997). The heme oxygenase system: a Regulator of Second Messenger Gases. Annu Rev Pharmacol Toxicol.

[21] Kennedy GY, Vevers HG (1973). Eggshell pigments of the Araucano fowl. Comp Biochem Physiol B..

[22] Lang MR, Wells JW (1987). A Review of Eggshell Pigmentation. Worlds Poult Sci J..

[23] Zakhary R, Gaine SP, Dinerman JL, Ruat M, Flavahan NA, Snyder SH (1996). Heme oxygenase 2: endothelial and neuronal localization and role in endothelium-dependent relaxation. Proc Natl Acad Sci.

[24] Zhao R, Xu GY, Liu ZZ, Li JY, Yang N (2006). A study on eggshell pigmentation: biliverdin in blue-shelled chickens. Poult Sci.

[25] Gorchein A, Lord G, Lim CK. Isolation and characterization of free haem from the shell gland of quail and hen. Biomed Chromatogr. 2011. 10.1002/bmc.1665 21678459

[26] Shetlar MD, Hill H (1985). Reactions of hemoglobin with phenylhydrazine: a review of selected aspects. Environ Health Perspect.

[27] Clark MW, Gildersleeve RP, Thaxton JP, Parkhurst CR, McRee DI (1988). Hematological effects of ethyl methanesulfonate, paraquat and phenylhydrazine in Japanese quail. Comp Biochem Physiol Part C Comp Pharmacol..

[28] Williams TD, Fronstin RB, Otomo A, Wagner E (2012). Validation of the use of phenylhydrazine hydrochloride (PHZ) for experimental manipulation of haematocrit and plasma haemoglobin in birds. Ibis..

[29] Fronstin RB, Christians JK, Williams TD (2015). Experimental reduction of haematocrit affects reproductive performance in European starlings. Funct Ecol.

[30] Christians JK, Williams TD (2001). Intraspecific variation in reproductive physiology and egg quality in the European Starling Sturnus vulgaris. J Avian Biol.

[31] Latunde-Dada GO, McKie AT, Simpson RJ (2006). Animal models with enhanced erythropoiesis and iron absorption. Biochim Biophys Acta.

[32] Fowler MA, Williams TD (2015). Individual variation in parental workload and breeding productivity in female European starlings: is the effort worth it?. Ecol Evol..

[33] Rowland E, Love OP, Verspoor JJ, Sheldon L, Williams TD (2007). Manipulating rearing conditions reveals developmental sensitivity in the smaller sex of a passerine bird, the European starling *Sturnus vulgaris*. J Avian Biol.

[34] Verspoor JJ, Love OP, Rowland E, Chin EH, Williams TD (2007). Sex-specific development of avian flight performance under experimentally altered rearing conditions. Behav Ecol.

[35] Love OP, Williams TD (2008). The adaptive value of stress-induced phenotypes: effects of maternally derived corticosterone on sex-biased investment, cost of reproduction, and maternal fitness. Am Nat..

[36] Wagner EC, Stables CA, Williams TD (2008). Hematological changes associated with egg production: direct evidence for changes in erythropoiesis but a lack of resource dependence?. J Exp Biol.

[37] Andersson S, Prager M. Quantifying colors. In: Bird coloration I: mechanisms and measurements. Cambridge: Harvard University Press; 2006. p. 41–89.

[38] Moreno J, Lobato E, Morales J, Merino S, Tomás G, la Puente JM, Sanz JJ, Mateo R, Soler JJ (2006). Experimental evidence that egg color indicates female condition at laying in a songbird. Behav Ecol.

[39] Morales J, Ruuskanen S, Laaksonen T, Eeva T, Mateo R, Belskii E, Ivankina EV, Järvinen A, Kerimov A, Korpimäki E, Krams I, Mänd R, Morosinotto C, Orell M, Qvarnström A, Siitari H, Slater FM, Tilgar V, Visser ME, Winkel W, Zang H, Moreno J (2013). Variation in eggshell traits between geographically distant populations of pied flycatchers *Ficedula hypoleuca*. J Avian Biol.

[40] López-Rull I, Miksik I, Gil D (2008). Egg pigmentation reflects female and egg quality in the spotless starling *Sturnus unicolor*. Behav Ecol Sociobiol.

[41] Falchuk KH, Contin JM, Dziedzic TS, Feng Z, French TC, Heffron GJ, Montorzi M (2002). A role for biliverdin IXα in dorsal axis development of Xenopus laevis embryos. Proc Natl Acad Sci.

[42] Burley RW, Vadehra DV (1989). The avian egg: chemistry and biology.

[43] Soh T, Fujihara N, Koga O (1993). Observations of pigment accumulation in the epithelium of the shell gland and superficial pigmentation on the eggshell in Japanese quail. J Fac Agric..

[44] Fernandez FR, Grindem CB, Feldman BF, Zinkl JG, Jain NC (2006). Reticulocyte response. Schalm’s veterinary hematology.

[45] De Coster G, De Neve L, Lens L (2012). Intraclutch variation in avian eggshell pigmentation: the anaemia hypothesis. Oecologia.

[46] Moreno J, Morales J, Lobato E, Merino S, Tomás G, la Puente JM (2005). Evidence for the signaling function of egg color in the pied flycatcher *Ficedula hypoleuca*. Behav Ecol.

[47] Morales J, Torres R, Velando A (2009). Parental conflict and blue egg coloration in a seabird. Naturwissenschaften.

[48] Stoddard MC, Fayet AL, Kilner RM, Hinde CA (2012). Egg speckling patterns do not advertise offspring quality or influence male provisioning in great tits. PLoS ONE.

[49] Cassey P, Ewen JG, Blackburn TM, Hauber ME, Vorobyev M, Marshall NJ (2008). Eggshell colour does not predict measures of maternal investment in eggs of *Turdus thrushes*. Naturwissenschaften.

[50] Butler MW, Waite HS (2016). Eggshell biliverdin concentration does not sufficiently predict eggshell coloration. J Avian Biol.

[51] Morales J, Sanz JJ, Moreno J (2006). Egg colour reflects the amount of yolk maternal antibodies and fledging success in a songbird. Biol Lett.

