# Supplementary material for: Haematocrit, eggshell colouration and sexual signaling in the European starling (Sturnus vulgaris)
Source: BMC Ecol. 2016 Jun 27;16:31. doi: 10.1186/s12898-016-0084-x (PMC4922052; doi:10.1186/s12898-016-0084-x)
Supplement: Supplementary file 1 — 10.1186/s12898-016-0084-x Traits of females that did or did not produce a replacement clutch. [file 12898_2016_84_MOESM1_ESM.docx]

Supplementary Table 1. Haematological and eggshell colour differences from the first (unmanipulated) clutch of females that were or were not observed to produce a replacement clutch.

| Term in model^1^ | Parameter | Pre-treatment Hct (%) | Pre-treatment Hb (g/dL) | Pre-treatment mean BGC |
| --- | --- | --- | --- | --- |
|  | N | 63 | 59 | 63 |
| Treatment x Replacement  Interaction | F | 2.50 | 0.68 | 0.24 |
|  | DF | 1, 59 | 1, 55 | 1, 59 |
|  | P | 0.12 | 0.41 | 0.62 |
| Treatment | F | 0.02 | 0.14 | 1.92 |
|  | DF | 1, 59 | 1, 55 | 1, 59 |
|  | P | 0.88 | 0.71 | 0.17 |
| Replacement | F | 0.16 | 1.03 | 0.12 |
|  | DF | 1, 59 | 1, 55 | 1, 59 |
|  | P | 0.69 | 0.32 | 0.73 |
| Observed to produce a replacement clutch | Least-squares mean ± standard error | 50.0 ± 0.7 | 14.2 ± 0.4 | 0.475 ± 0.002 |
| Not observed to produce a replacement clutch | Least-squares mean ± standard error | 50.4 ± 0.6 | 14.7 ± 0.3 | 0.475 ± 0.002 |

^1^A general linear model was performed including effects of treatment (PHZ or saline), replacement (whether or not female was observed to produce a replacement clutch), and the interaction between treatment and replacement.
