# Supplementary material for: Haematocrit, eggshell colouration and sexual signaling in the European starling (Sturnus vulgaris)
Source: BMC Ecol. 2016 Jun 27;16:31. doi: 10.1186/s12898-016-0084-x (PMC4922052; doi:10.1186/s12898-016-0084-x)
Supplement: Supplementary file 2 — 10.1186/s12898-016-0084-x Differences between treatment groups, including laying interval in the model. [file 12898_2016_84_MOESM2_ESM.docx]

Supplementary Table 2. Post-treatment haematological and eggshell colour differences between treatment groups, including laying interval in the model.

| Trait | N | F | Df | P | PHZ^1^ | Saline^1^ | Additional terms in model |
| --- | --- | --- | --- | --- | --- | --- | --- |
| Post-treatment Hct (%) | 29 | 0.05 | 1,25 | 0.82 | 51.6 ± 1.2 | 51.2 ± 1.0 | Pre-treatment Hct, F_1,25_ = 3.11, P = 0.09; Laying interval F_1,25_ = 0.76, P = 0.39 |
| Post-treatment Hb (g/dL) | 26 | 4.57 | 1,22 | 0.04 | 17.3 ± 0.6 | 15.6 ± 0.5 | Pre-treatment Hb, F_1,22_ = 6.53, P = 0.018; Laying interval F_1,22_ = 0.04, P = 0.84 |
| Post-treatment mean BGC | 28 | 0.33 | 1,24 | 0.57 | 0.470 ± 0.002 | 0.468 ± 0.002 | Pre-treatment BGC, F_1,24_ = 15.21, P = 0.0007; Laying interval F_1,24_ = 0.83, P = 0.37 |
| BGC of first egg of replacement clutch | 20 | 0.13 | 1, 16 | 0.72 | 0.466 ± 0.003 | 0.468 ± 0.002 | Pre-treatment BGC, F_1,16_ = 17.15, P = 0.0008; Laying interval F_1,16_ = 0.42, P = 0.53 |

^1^Means are least-squares means ± standard errors from general linear models.
