# Supplementary material for: Haematocrit, eggshell colouration and sexual signaling in the European starling (Sturnus vulgaris)
Source: BMC Ecol. 2016 Jun 27;16:31. doi: 10.1186/s12898-016-0084-x (PMC4922052; doi:10.1186/s12898-016-0084-x)
Supplement: Supplementary file 3 — 10.1186/s12898-016-0084-x Statistical interactions between eggshell colour and treatment. [file 12898_2016_84_MOESM3_ESM.docx]

Supplementary Table 3. Associations between eggshell colour and reproductive performance in replacement clutches.

|  |  | Mean BGC | |  | Treatment | |  | Mean BGC * Treatment | |
| --- | --- | --- | --- | --- | --- | --- | --- | --- | --- |
| Trait | df | F | P |  | F | P |  | F | P |
| Interval between treatment and day first egg of replacement clutch (days) | 1,24 | 0.00 | 0.95 |  | 0.01 | 0.93 |  | 0.02 | 0.90 |
| Mean egg mass | 1,24 | 0.20 | 0.66 |  | 2.85 | 0.10 |  | 2.95 | 0.10 |
| Clutch size | 1,24 | 1.06 | 0.31 |  | 0.18 | 0.68 |  | 0.20 | 0.66 |
| Female body mass at clutch completion | 1,24 | 0.70 | 0.41 |  | 0.26 | 0.61 |  | 0.27 | 0.60 |
| Brood size at hatch | 1,24 | 0.15 | 0.70 |  | 3.54 | 0.07 |  | 3.57 | 0.07 |
| Hatchling mass^1^ | 1,23 | 0.30 | 0.59 |  | 0.64 | 0.43 |  | 0.69 | 0.41 |
| Maternal provisioning (nest visits per chick) | 1,22 | 0.63 | 0.44 |  | 0.31 | 0.58 |  | 0.28 | 0.60 |
| Paternal provisioning (nest visits per chick) | 1,22 | 0.67 | 0.42 |  | 3.36 | 0.08 |  | 3.33 | 0.08 |
| Total provisioning (nest visits per chick) | 1,22 | 0.63 | 0.44 |  | 1.22 | 0.28 |  | 1.17 | 0.29 |
| Hct of 17 day old chicks^1^ | 1,19 | 1.57 | 0.22 |  | 1.63 | 0.22 |  | 1.52 | 0.23 |
| Hb of 17 day old chicks^1^ | 1,19 | 5.78 | 0.03 |  | 0.23 | 0.63 |  | 0.25 | 0.62 |
| Mean tarsus length of 17 day old chicks^1^ | 1,22 | 4.16 | 0.054 |  | 0.11 | 0.75 |  | 0.10 | 0.75 |
| Mean mass of 17 day old chicks^1^ | 1,22 | 1.07 | 0.31 |  | 0.11 | 0.75 |  | 0.10 | 0.76 |
| Brood size at fledging | 1,24 | 1.06 | 0.31 |  | 1.29 | 0.27 |  | 1.30 | 0.27 |

Results are from general linear models. Although 29 females produced a replacement clutch, eggshells were not recovered from one replacement clutch and so sample sizes are 28 for all traits except provisioning, for which they are 26.

^1^ Repeated measures analysis with female identity included as a repeated subject effect. The analysis of hatchling mass included first clutch egg mass as a covariate (F_1,23_ = 64.60, P < 0.0001) since eggs were larger pre-treatment in the PHZ group.
